# A Multiscale Approach to Characterize the Early Aggregation Steps of the Amyloid-Forming Peptide GNNQQNY from the Yeast Prion Sup-35

**DOI:** 10.1371/journal.pcbi.1002051

**Published:** 2011-05-19

**Authors:** Jessica Nasica-Labouze, Massimiliano Meli, Philippe Derreumaux, Giorgio Colombo, Normand Mousseau

**Affiliations:** 1 Département de Physique and GEPROM, Université de Montréal, Montréal, Québec, Canada; 2 Istituto di Chimica del Riconoscimento Molecolare, CNR, Milano, Italy; 3 Laboratoire de Biochimie Théorique, UPR9080 CNRS, Institut de Biologie Physico-Chimique, Université Paris 7, and Institut Universitaire de France, Paris, France; Stanford University, United States of America

## Abstract

The self-organization of peptides into amyloidogenic oligomers is one of the key events for a wide range of molecular and degenerative diseases. Atomic-resolution characterization of the mechanisms responsible for the aggregation process and the resulting structures is thus a necessary step to improve our understanding of the determinants of these pathologies. To address this issue, we combine the accelerated sampling properties of replica exchange molecular dynamics simulations based on the OPEP coarse-grained potential with the atomic resolution description of interactions provided by all-atom MD simulations, and investigate the oligomerization process of the GNNQQNY for three system sizes: 3-mers, 12-mers and 20-mers. Results for our integrated simulations show a rich variety of structural arrangements for aggregates of all sizes. Elongated fibril-like structures can form transiently in the 20-mer case, but they are not stable and easily interconvert in more globular and disordered forms. Our extensive characterization of the intermediate structures and their physico-chemical determinants points to a high degree of polymorphism for the GNNQQNY sequence that can be reflected at the macroscopic scale. Detailed mechanisms and structures that underlie amyloid aggregation are also provided.

## Author Summary

The formation of amyloid fibrils is associated with many neurodegenerative diseases such as Alzheimer's, Creutzfeld-Jakob, Parkinson's, the Prion disease and diabetes mellitus. In all cases, proteins misfold to form highly ordered insoluble aggregates called amyloid fibrils that deposit intra- and extracellularly and are resistant to proteases. All proteins are believed to have the instrinsic capability of forming amyloid fibrils that share common specific structural properties that have been observed by X-ray crystallography and by NMR. However, little is known about the aggregation dynamics of amyloid assemblies, and their toxicity mechanism is therefore poorly understood. It is believed that small amyloid oligomers, formed on the aggregation pathway of full amyloid fibrils, are the toxic species. A detailed atomic characterization of the oligomerization process is thus necessary to further our understanding of the amyloid oligomer's toxicity. Our approach here is to study the aggregation dynamics of a 7-residue amyloid peptide GNNQQNY through a combination of numerical techniques. Our results suggest that this amyloid sequence can form fibril-like structures and is polymorphic, which agrees with recent experimental observations. The ability to fully characterize and describe the aggregation pathway of amyloid sequences numerically is key to the development of future drugs to target amyloid oligomers.

## Introduction

The aggregation of soluble peptides and proteins first into soluble oligomeric assemblies and then into insoluble amyloid fibrils is associated with the onset of misfolding diseases such as Alzheimer's disease, Parkinson's disease, type II diabetes and transmissible spongiform encephalopathies [1]–[5]. Though there is no sequence similarity, the final products of all amyloidogenic proteins display a similar cross-β structure [6], [7] and the soluble oligomers of several proteins appear to share similar structural properties [8], suggesting common pathways for amyloid formation [8]–[10]. Structural similarity does not, however, exclude diversity or polymorphism in the intermediates and products of amyloid assembly [11]–[24].

Many studies have shown that soluble oligomeric intermediates are more toxic than the full fibrils themselves [25], [26]. These transient oligomers include low molecular weight aggregates (e.g. dimers [27] and tetramers [28]) and high molecular weight species (e.g., β-sheet rich annular protofibrils similar to pore-forming toxins [29]–[32]). While oligomers are considered as primary toxic species for most neurodegenerative diseases, there is recent experimental evidence that fragmentation or breakage of fibrils can contribute to the kinetics of aggregation and the amyloid cytotoxicity itself [33], [34].

One important way for investigating amyloid fibril formation, polymorphism and cytotoxicity is offered by short protein fragments. Among them, GNNQQNY, from the N-terminal prion-determining domain of the yeast protein Sup35, is a paradigmatic example of a short sequence with the same properties as its corresponding full-length protein [35], [36]. These properties include an amyloid fibril with a core cross-β spine, Congo-red binding and a nucleated-growth aggregation process [35]. In particular, X-ray diffraction of several micro-crystals provides a detailed atomic structure for different GNNQQNY fibrillar morphologies where the side-chains form self-complementing steric zippers [6], [7], [35]–[37]. As for all amyloid sequences, structural characterization of the intermediate GNNQQNY oligomers has been however precluded experimentally due to the high complexity of the aggregation process, and the short-lived and meta-stable character of the early aggregates.

Computer simulations have proved useful complements to experiments for looking at the initial aggregation steps providing information, for example, about the presence of amorphous states in dynamic equilibrium with fibrillar and annular states [38]–[41] and the final steps of the polymerization-nucleation process [23], [42]. They can provide atomic-resolution insights into several factors, ranging from the effect of sequence variations on aggregation tendencies to information on the stability of aggregates and the kinetics of aggregation. Due to lighter computational costs, short peptides are more amenable to simulations of the aggregation process than full-length proteins. For example, a number of numerical works have characterized the structures and free energy of small GNNQQNY aggregates ranging from 2-mers to 8-mers starting from disordered states or studied the stability of pre-formed GNNQQNY assemblies with cross-β or annular morphologies [23], [24], [32], [42]–[51].

In this paper, we push the boundaries of the GNNQQNY oligomer size and investigate, through a multi-scale simulation approach, the aggregation and polymorphism of three GNNQQNY oligomer sizes: 3-mers, 12-mers and 20-mers. Our approach takes advantage of the accelerated sampling properties of replica exchange molecular dynamics (REMD) simulations [52] based on coarse-grained models and of the accurate description of the physico-chemical interactions between the peptides and the solvent by using an all-atom model. More precisely, we first use REMD simulations [52] with the coarse-grained potential OPEP [53], [54], and then analyze the stability and conformational properties of selected aggregates by room temperature MD as well as REMD simulations using the GROMOS force-field [55]. In total, we accumulated more than 23.60 µs and 2.66 µs of coarse-grained and all-atom simulations, respectively, allowing relevant statistical analysis. To our knowledge, the present study reports the largest simulations of spontaneous self-organization carried out at the atomic resolution on an amyloid peptide without any pre-formed seed. Overall, the results of our integrated simulations and analysis show the existence of a high degree of polymorphism for the GNNQQNY sequence, even for oligomeric assemblies containing as many as 20 monomers.

## Materials and Methods

Simulations and analyses presented here couple a number of approaches, which are described briefly in this section. The first set of simulations uses the coarse-grained OPEP potential with replica-exchange molecular dynamics (REMD). These are followed by all-atom simulations using GROMACS with MD and REMD. All simulations are labeled as follows: a number, which indicates the number of monomers, two letters indicating the force field (OP for OPEP and GR for GROMACS), a letter or number indicating the simulation and a label for the specific conformation studied (when appropriate) giving, for example: 01OP2-A1.

### Replica-Exchange Molecular Dynamics (REMD)

REMD is a thermodynamical sampling method that requires the running of N MD trajectories (or replica) in parallel at N different temperatures selected in order to optimize thermodynamical sampling [52]. At regular time intervals, conformational exchanges are attempted between adjacent simulation pairs according to the Metropolis criterion with accept-reject probability:
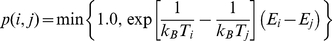
where, before the exchange, trajectory *i* at temperature *T_i_* has an energy *E_i_* and trajectory *j* has an energy *E_j_* at temperature *T_j_*.

This broadly used method allows for conformations in a deep local minimum to explore other regions of the energy landscape by migrating to higher temperatures. While thermodynamical properties converge faster than with single temperature standard MD, dynamical information is lost due to temperature exchanges. It is still possible, however, to derive thermodynamically putative aggregation pathways by following the continuous trajectories through temperature space.

### The Optimal Potential For Efficient Peptide-Structure Prediction (OPEP) Force-Field

OPEP is a coarse-grained protein model that uses a detailed representation of all backbone atoms (N, H, Cα, C and O) and reduces each side-chain to one single bead with appropriate geometrical parameters and van der Waals radius. The OPEP energy function, which includes implicit effects of aqueous solution, is expressed as a sum of local potentials (taking into account the changes in bond lengths, bond angles, improper torsions of the side-chains and backbone torsions), non-bonded potentials (taking into account the hydrophobic and hydrophilic properties of each amino acid) and hydrogen-bonding potentials (taking into account two- and four- body interactions) [53]. OPEP has been extensively tested on peptides using multiple approaches such as the activation-relaxation technique [56], Monte Carlo [54], MD [39] and REMD simulations [57], and greedy-based algorithms [58], [59]. OPEP is also appropriate for simulations of GNNQQNY. Preliminary test simulations on this peptide's dimer indicate that, at 300 K, the GNNQQNY relative orientation is a 60 to 40 probability in favor of the antiparallel dimer with a least two hydrogen bonds. This result is in general agreement with what was found by Strodel *et al.* with CHARMM19 and the implicit solvation potential EEFI [46] where both orientations of the strands are visited with similar probabilities.

### OPEP Simulation Details

**Figure 1:**
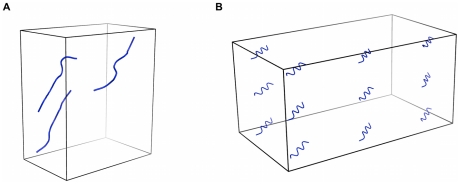
Starting structures for a) the trimer and b) dodecamer. The concentration for both systems is set at 4.15 mM. The random coil monomers are placed 15 Å apart.

**Figure 2:**
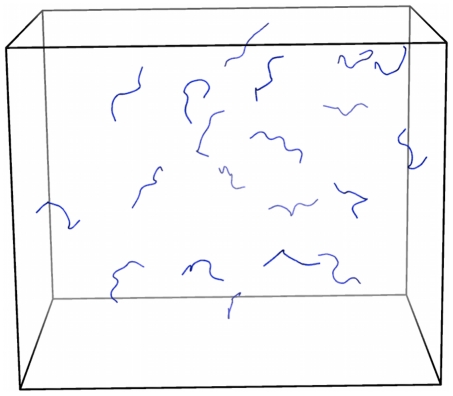
Starting structure with random coils and no seed for the 20-mer simulations. The concentration is also 4.15 mM. The monomers are randomly placed 12 to 50 Å apart.

REMD were carried out using a 1.5 fs time-step, periodic boundary conditions with box sizes depending on the systems and a weak coupling to an external temperature bath [60], [61], [62]. Replica exchanges were attempted every 5000 steps and configurations saved every 5000 steps. Initial structures for 3-mer and the 20-mer simulations were constructed by placing random coil monomers between 12 Å to 50 Å apart (**Figure 1a** **and** **Figure 2**). For the 12-mer, the initial chains occupied four rows, with each peptide separated from the others by 15 Å (**Figure 1b**). Because of the extensive sampling of REMD, all results are independent from this initial setup. For the 20-mer system, three OPEP-REMD simulations were launched. A preliminary REMD simulation (20OPp) was used to obtain a first estimate of the melting temperature (Tm = 283 K) and generate some representative conformations for all-atom MD refinement. The 20 initial temperatures were logarithmically distributed between 230 K and 450 K. Despite 200-ns simulation per replica, we found that the configuration space was not optimally sampled because of the existence of a large discontinuity in the potential energy when the system orders. Thus, a Gaussian distribution of temperatures around 283 K was deemed preferable to allow a better sampling of the phase space. The other two REMD simulations, running for 400 ns at each temperature, were started from the same random configuration (**Figure 2**), but with an optimized Gaussian temperature distribution centered around 283 K: 20OP1 uses 20 temperatures (in Kelvins: 223.8, 249.2, 260.1, 266.0, 270.3, 273.8, 277.1, 280.1, 283.0, 285.9, 288.9, 292.2, 295.7, 300.1, 305.9, 316.8, 342.2, 370.1, 398.0, 426.0) and 20OP2 uses 22 temperatures, with two more temperatures below the transition, at 236.5 and 254.7 K, to increase exchanges between low-energy structures. All REMD simulations are summarized in **Table 1**.

**Table 1:** Details of all simulations run for the trimer, dodecamer and 20-mer systems.

	Length of OPEP simulations (ns)(a)	OPEP - LABEL of structures extracted(b)	Temperatures min-max (K) & number of temperatures(c)	GROMACS - LABEL of reconstructed OPEP extracted structures(b)	Length of GROMACS simulations (ns)(d)	Total number of atoms(e)	Temperature (K)(f)
3-mer	50×16	03OP1-A	222.5–525 16	03-GR1-A	100	6504	300
		03OP1-B	222.5–525 16	03-GR1-B	100	5997	300
		03OP1-C	222.5–525 16	03-GR1-C	100	5619	300
		03OP1-D	222.5–525 16	03-GR1-D	100	5949	300
		03OP1-E	222.5–525 16	03-GR1-E	100	6621	300
12-mer	125×16	12OP1-A	222.5–525 16	12-GR1-A	100	56271	300
		12OP1-B	222.5–525 16	12-GR1-B	100	40476	300
		12OP1-C	222.5–525 16	12-GR1-C	100	15381	300
		12OP1-D	222.5–525 16	12-GR1-D	100	16134	300
		12OP1-E	222.5–525 16	12-GR1-E	100	17157	300
20-mer OPp	200×20	20OPp-A	234.6–447.6 20	20GRp-A1	100	25620	300
		20OPp-A	234.6–447.6 20	20GRp-A2	100	25620	300
		20OPp-B	234.6–447.6 20	20GRp-B1	100	27816	300
		20OPp-B	234.6–447.6 20	20GRp-B2	100	27816	300
		20OPp-B	234.6–447.6 20	20GRp-B3 (REMD)	10×12	27816	see text
		20OPp-C	234.6–447.6 20	20GRp-C1	100	49674	300
		20OPp-C	234.6–447.6 20	20GRp-C2	100	49674	300
		20OPp-D	234.6–447.6 20	20GRp-D1	100	23746	300
		20OPp-D	234.6–447.6 20	20GRp-D2	100	23746	300
20-mer OP2	400×22	20OP2-A	223.8–425.9 22	20GR2-A	100	21586	300
		20OP2-B	223.8–425.9 22	20GR2-B	100	24640	300
		20OP2-C	223.8–425.9 22	20GR2-C	100	23554	300
		20OP2-E	223.8–425.9 22	20GR2-E	100	26065	300
		20OP2-N	223.8–425.9 22	20GR2-N1	100	32206	300
		20OP2-N	223.8–425.9 22	20GR2-N2 (REMD)	10×12	32206	see text

This table presents simulations done with OPEP (coarse-grained potential) and GROMACS (all-atom potential).

(a) The total simulation time for OPEP REMD simulations in the format time_per_replica x number_of_replicas.

(b) The label of the OPEP/GROMACS structures extracted. The label indicates the number of monomers, the potential used (OP for OPEP and GR for GROMACS), the simulation index (1,2 or p (preliminary)) and the letter ID of the structure.

(c) The range of temperatures (in K) used for OPEP REMD simulations.

(d) The total simulation time for GROMACS simulations. MD simulations are indicated by only one number while, for REMD simulations, the total simulation time is given in the format time_per_replica x number_of_replicas.

(e) The total number of atoms in the system including protein and solvatation water atoms.

(f) The temperature used in GROMACS simulations (in K).

Determining whether equilibrium has been reached, even for the trimer, is difficult. It is always possible that a system is stuck in a minimum and thermodynamical properties will then appear as though they are converged. Here, we use the specific heat to track convergence. This quantity, the second derivative of the free energy, is very sensitive to convergence at all temperatures, and provides a very stringent test even near transitions. Because we are mostly interested in the qualitative properties of the systems under study here, we consider that a system is converged when the overall shape of the specific heat near the transition is converged. This ensures that the dominant structures are found with the proper weight, within the limits of our simulations.

### OPEP Analysis and Structure Selection

Analysis for these simulations was performed, in part, using a new clustering code that enables us to identify the dominant configuration types in terms of clusters formed in β-sheet structures based on strand attachment. The criterion set to define a hydrogen bond between two given strands is similar to the one used in the DSSP algorithm. [63]. A cutoff of one hydrogen bond is used for distinguishing random from β-strands since we are dealing with a very short sequence and not considering the hydrogen bonds with the N-terminal glycines. The configuration types are defined here in terms of the number of sheets and the number of strands per sheet in the structure. For instance, a configuration type 8 7 5 for the 20-mer describes a structure with 3 β-sheets containing 8, 7 and 5 strands, respectively. The clustering code also provides information about the orientation of the strands in a β-sheet (i.e., parallel or anti-parallel), alignment of the β-strands within a β-sheet (i.e., in register or out-of register) and nature of the β-sheets (i.e. fully parallel, full anti-parallel or mixed orientations within a sheet). In addition to the clustering analysis, a PTWHAM analysis [64] was also performed on all of our data to compute thermodynamical properties.

In all cases, structures for all-atom simulations were taken among those of lower-energy OPEP that resisted most efficiently to a temperature increase during replica exchanges. For one preliminary simulation (20OPp) however, the structures were selected based on their frequency of occurrence.

### All-Atom MD Analysis of the Conformational and Stability Properties of Opep-Generated, Selected Oligomeric Structures

The initial structures for all-atom, explicit solvent Molecular Dynamics (MD) simulations were built by reconstructing the atomic detail of selected conformations from the OPEP coarse-grained runs. Reconstruction was carried out using the MAXSPROUT server [65]. Refinement of side-chain rotameric states was performed using the program IRECS [66], [67], where the prediction is guided by a combination of potential interaction and rotamer scores calculated with probabilities from the backbone dependent rotamer library. Resulting all-atom structures obtained with this procedure were first minimized using the Macromodel package (Schrodinger Incorporated, USA) for 5000 steps with Polak-Ribier Conjugate Gradient method and an energy gradient criterion for convergence set to 0.05 kJ/mol. This minimization protocol was intended to initially remove unphysical contacts between atoms resulting from the reconstruction procedure, and not to optimize structures. At this stage, the Cα atoms were constrained to their positions with the default force constant (25 kcal/mol Å^2^).

The resulting minimized systems were then solvated in a cubic-shaped box large enough to contain 1.0*nm* of solvent around each initial aggregate. The simple point charge (SPC) water model was used [68] to solvate each oligomer in the simulation box. Each system was subsequently energy minimized with a steepest descent method for 5000 steps. The minimization was considered to be converged when the maximum force was smaller than 0.0001 kJ mol^−1^ nm^−1^. The initial step size for minimization was 0.01 nm. The calculation of electrostatic forces was done with the PME implementation of the Ewald summation method. The LINCS [69] algorithm was used to constrain all bond lengths and the SETTLE algorithm [70] for the water molecules. Simulations were performed with a dielectric permittivity, 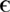 = 1, and a time step of 2 fs. Initial velocities were taken from a Maxwellian distribution at the desired initial temperature of 300 K. The density of the system was adjusted performing the first equilibration runs at NPT condition by weak coupling to a bath of constant pressure (P_0_ = 1 bar, coupling time τ*_P_* = 0.5 ps) [60] and the system was weakly coupled to an external temperature bath [60] with a coupling constant of 0.1 ps. The proteins and the rest of the system were coupled separately to the temperature bath. Table 1 summarizes the simulation conditions and number of peptides for each simulation. All simulations and analysis were carried out using the GROMACS package (version 3.3) [71]–[73] and the GROMOS96 43A1 force field [74]–[77].

For all MD simulations, aggregates were simulated at 300 K for 100 ns. REMD simulations were also used to investigate the stability and the conformational preferences of two 20-mer aggregates. The replica exchange simulations were carried out using the Solute Tempering REMD [78] protocol using the version implemented in GROMACS by de Groot and coworkers [79]. Twelve temperatures between 308 K and 419 K were selected according to [80] for an exchange probability of around 40%.

## Results/Discussion

The aggregation process for the three types of GNNQQNY oligomers – containing 3, 12 and 20 chains, respectively – was studied by a multi-scale approach consisting in a preliminary, thorough exploration of the phase space through REMD with the OPEP coarse-grained potential, followed by the refinement of the most representative aggregate structures obtained via all-atom MD or REMD simulations in explicit solvent. The initial concentration for the OPEP runs was around 4.15 mM. This concentration is 10 times higher than the concentration at which amyloid GNNQQNY fibrils form in a few hours according to Nelson et al. [9] allowing for the formation of ordered structures within our simulation time frame. The diversity in the number of chains allows us to examine possible intermediates and analyze molecular mechanisms of polymorphism in amyloid aggregates.

For clarity, we first present and discuss results for the trimeric and dodecameric systems as they will serve as basis for understanding the results observed for the 20-mer presented in the last part of this section.

### Simulations of Trimeric Systems

#### Coarse-grained simulations

**Figure 3:**
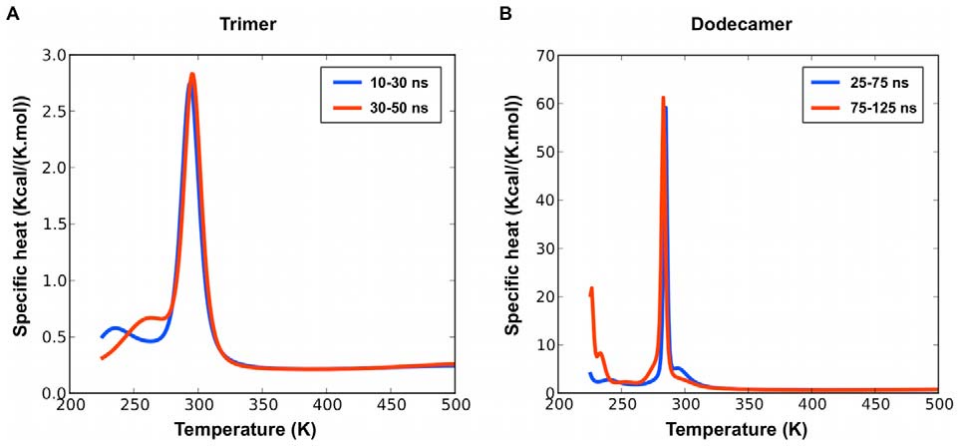
Specific heat as a function of temperature for the trimer and dodecamer systems. The specific heat is calculated over two time intervals for each system (trimer on the left panel and dodecamer on the right panel). Both systems have converged over the time windows displayed here.

Coarse-Grained REMD simulations were performed with 16 replicas for 50 ns at temperatures discussed in the materials and methods section. Although the system is not fully converged for the very low-temperature replicas, the PTWHAM-generated specific heat computed over two different time intervals shows that the melting temperature, T_m_, is well-established at ∼294 K (**Figure 3**). Below this temperature, a clustering analysis shows that GNNQQNY monomers are assembled into ordered structures with high β-sheet content, while above T_m_, the system visits mostly disordered structures with very low secondary structure composition. The alignment of individual strands within oligomers, the secondary structures and the configuration types of the aggregates are summarized in **Table 2**.

**Table 2:** Structural characteristics for small aggregates as a function of temperature.

			Population						
			222.5 K	235.8 K	250.8 K	266.7 K	283.4 K	300 K	313.8 K
Trimer	Configuration types (%)(a)	3	96.3	100	98.2	98.2	92.6	13	1.9
		2 1	3.7	0	1.8	1.8	1.9	22.2	9.2
		1 1 1	0	0	0	0	5.5	64.8	88.9
	% parallel(b)		45.4	39.8	22.2	12	6.5	7.4	0
	% antiparallel(b)		54.6	60.2	77.8	88	88	24.1	5.6
	% fully parallel sheets(c)		0	0	1.9	0	0	21.1	0
	% fully antiparallel sheets(c)		9.3	20.4	57.4	75.9	86.3	68.4	50
	% mixed sheets(c)		90.7	79.6	40.7	24.1	13.7	10.5	50
	β-sheet content (%)(d)		72.4	70.6	66.6	61.6	55.1	33.3	28.3
	% Strands in-register/out-of-register by 1 residue(e)		51.9/46.3	47.2/48.2	47.2/38.9	38.0/43.5	17.7/45.1	21.1/42.1	33.3/41.7
Dodecamer	Configuration types (%)(a)	7 5	0	4.5	9.8	54.1	75.2	34.6	0
		7 4 1	30.8	33.1	12.8	1.5	0.8	1.5	0
		8 4	0	11.3	38.4	36.8	5.3	1.5	0
		12	66.9	7.5	9	0.8	1.5	0.8	0
		11 1	2.3	15	9	3	1.5	0.8	0
		10 1 1	0	9	6	0	3	1.5	0
	% parallel(b)		32.3	31.3	40.9	47.9	41.9	37.9	17.1
	% antiparallel(b)		60.2	48.4	45.8	46.7	49.5	41.1	16
	% fully parallel sheets(c)		0	0.4	1.2	0.4	0.8	15.3	22.6
	% fully antiparallel sheets(c)		6.3	10.8	6.2	2.3	2.7	11.4	21.8
	% mixed sheets(c)		93.7	88.8	92.6	97.3	96.5	73.3	55.6
	β-sheet content (%)(d)		75.8	59.1	61.7	63.5	61.8	43.8	11.4
	% Strands in-register/out-of-register by 1 residue(e)		52.5/27.1	33.5/46.3	39.2/41.7	44.7/43.4	38.8/54.0	30.6/45.6	29.5/39.7

Temperatures above 313.8 K are not displayed here since they are populated essentially by conformations with random coil monomers with no secondary structure. The percentages are calculated over all the structures obtained in the last 40 ns (trimer) and in the last 100 ns (dodecamer) of the OPEP REMD simulations, where the systems have converged.

(a) The dominant configuration types (as described in the OPEP Analysis and Structure Selection section).

(b) The average amount of parallel and anti-parallel strands in the β-sheets formed. The sum of parallel and antiparallel strands in a structure does not always total 100% if the structure sees strands in an undefined orientation, i.e. attached by only one hydrogen bond.

(c) The average amount of fully parallel, fully antiparallel and mixed sheets.

(d) The average amount of residues in a β conformation.

(e) The average amount of strands in-register and out-of-register (by one residue) in β-sheets.

**Figure 4:**
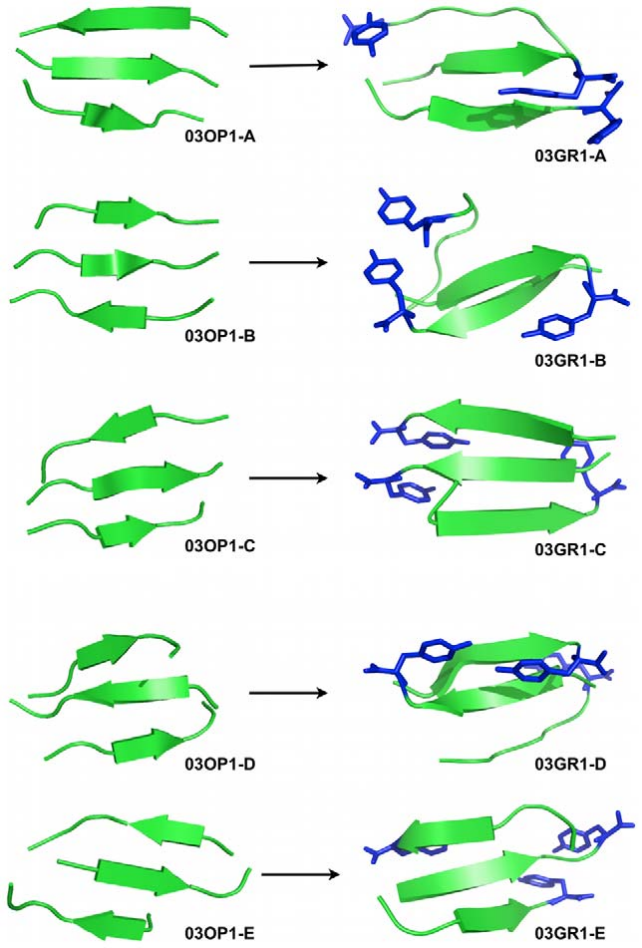
Structures obtained for the trimeric simulations. We show, on the left-hand side panel, representative structures obtained from the OPEP simulations and, on the right-hand side panel, the representative structures obtained after all-atom MD refinements. 03OP1-A,-B,-C,-D and –E were extracted respectively at 222.5 K (probability of occurrence for this β-strand organization: 91%), 235.7 K (80%), 250.8 K (41%), 266.7 K (76%) and 283.4 K (86%). 03OP1-A to -C are mixed β-sheets while 03OP1-D and –E are fully antiparallel β-sheets. The all-atom structures are represented in secondary structure cartoon and only the tyrosines (most hydrophobic residues in the sequence) are shown in blue sticks (hydrogen atoms are omitted).

Structurally, the trimer displays a strong tendency to form ordered planar β-sheets below T_m_ (**Figure 4** **, left part of the panel**). These appear rapidly, within 1 to 8 ns, in a mostly anti-parallel organization. Following trajectories leading to ordered structures, we see that the three-stranded β-sheet is always preceded by the formation of a mostly anti-parallel dimer seed. Averaging over all structures below T_m_, we find that only a very small proportion of structures just below T_m_ consist of a two-stranded β-sheet interacting with one chain in coil conformation (1.9%) or three random coil chains (1.1%). The peptides at a temperature just below T_m_ prefer an anti-parallel β-strand order (87% at 267 K) over a parallel arrangement (13% at 267 K), while this proportion falls to 55–60% at the lowest temperatures. As seen in **Table 2**, the three β-strands prefer to be perfectly aligned or in-registered at the lowest temperatures and are typically shifted by one residue, i.e. out-of-registered, at temperatures close to the melting point. As the temperature increases, the population of two-stranded and three-stranded β-sheets becomes very low, amounting to 8% and 0% at 333 K and 352 K, respectively. Except for the lowest temperatures, where mixed parallel/antiparallel sheets are most common, there is a clear dominance of fully antiparallel sheets for three-stranded structures while fully parallel sheets are rare, even among the few three-stranded sheets found above T_m_, where they reach 21%, to 68% for fully anti-parallel.

#### All-atom MD simulations

Five representative OPEP-generated structures, labeled 03OP1-A, 03OP1-B, 03OP1-C, 03OP1-D and 03OP1-E (**Figure 4** **, left side of the panel**), were then subjected to all-atom MD simulations as described in materials and methods. These structures can be divided in two sets: 03OP1-A, 03OP1-B, 03OP1-C are characterized by three-stranded β-sheets with mixed parallel/anti-parallel β-strands, while 03OP1-D and 03OP1-E display a fully anti-parallel three-stranded β-sheet.

As seen in the final structures of the all-atom simulations displayed in Figure 4 (right side of the panel), the five structures show different evolutions after the 100 ns all-atom MD. The three structures 03OP1-A, -B and -D tend towards configuration types 2-1, i.e. with one chain converted from β-strand to random coil and the two other chains enhancing their β-sheet contents. This inter-conversion is independent on the initial orientation of the strands. In contrast, the other two structures 03OP1-C and –E preserve their three-stranded β-sheet configurations and enhance their β-sheet contents. Simulation 03GR1-C keeps its starting mixed parallel/anti-parallel configuration of the strands; in the simulation 03GR1-E, one of the peptide flips orientation leading to a perfectly aligned mixed β-sheet from an initial fully anti-parallel sheet.

Even though all-atom simulations cannot capture fully disordered chains within 100 ns at 300 K, the coarse-grained and all-atom simulations indicate that both parallel and anti-parallel arrangements can be found in multiple meta-stable two-stranded and three-stranded structures, with various registers of hydrogen bonds contributing to the structural richness and conformational variability of the trimeric aggregates.

Our trimeric results point to the existence of three minima associated with parallel, antiparallel and mixed parallel/antiparallel β-sheet structures, and are consistent with previous computational studies at the all-atom level on the GNNQQNY trimer [44], [45], [51]. Our conformational distribution for the trimer is not biased, therefore, from the use of the OPEP coarse-grained potential. We emphasize that the population of the fully parallel and antiparallel β-structures in small aggregates vary substantially with the selected force field. Using CHARMM force field and the EEF1 implicit water model, Wales *et al.* predicted equal populations for both states from free energy calculations [46]. Lai *et al.* using multiple MD simulations with the Gromos force field and the SPC explicit water models found many transitions between both states [45], while Reddy *et al.* using the same Gromos force field and the SPC explicit water model predicted a much higher population for the parallel geometry [47].

### Simulations of Dodecameric Systems

#### Coarse-grained simulations

OPEP-REMD was performed with the 16 replicas as in the case of the trimer, but each for 125 ns. Within the first 25 ns, the system converges at low temperature to β-sheet rich structures where the strands prefer an antiparallel orientation, as for the trimer, but with a lower melting temperature of 283 K (see **Figure 3**) even though the potential energy per monomer in the ordered phase is much lower, reaching −37.0 kcal/mol/monomer for the 12-mer compared to −18.4 kcal/mol/monomer for the trimer, indicating a clear bias toward aggregation and resulting in a much more marked peak in the specific heat.

**Figure 5:**
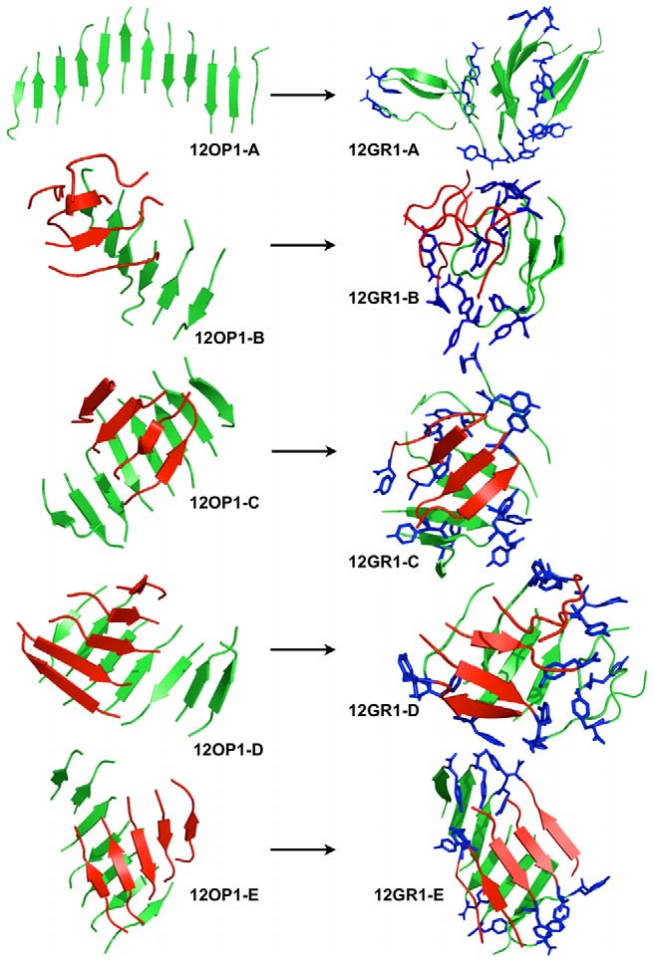
Structures obtained for the dodecameric simulations. We show, on the left-hand side panel, representative structures obtained from the OPEP simulations and, on the right-hand side panel, representative structures obtained after all-atom MD refinements. 12OP1-A,-B,-C,-D and –E were extracted respectively at 222.5 K, 235.7 K, 250.8 K, 266.7 K and 283.4 K. 12OP1-A (top left structure) is a long flat beta-sheet. 12OP1-B to -E (second left to bottom left structures) are made of 2 beta-sheets facing each other. Monomers forming β-sheets in the initial state are colored red or green. These colors are kept in the final structure. The tyrosines are shown in blue sticks for the all-atom structures. During the all-atom MD simulation the structures tend to be more globular but the strands see no exchange between the β-sheets, i.e. the red and green β-sheets do not dissociate for the 12-mer system.

Kinetically, the aggregation tendency for the dodecamer is to first form one or two stable four-stranded β-sheets that show little dissociation and that trigger the transient formation of one or two longer β-sheets. The formation of a trimer that precedes the four-stranded β-sheet shows, however, a higher dissociation/association rate. Interestingly, the tendency of the GNNQQNY sequence to form stable tetrameric aggregation nuclei had already been noticed in a previous investigation on the system [44] and was proposed by the Eisenberg group on the basis of entropic and energetic arguments [7]. The final stable ordered structures are shown in **Figure 5** (left side of the panel).

As would be expected, a rich set of ordered configurations is visited for the 12-mer (**Table 2**). Regrouping all structures below melting, the dominant conformation, visited 63% of the time, is a two β-sheet structure with a 7 or 8-strand sheet stabilized by a smaller, 4–5 strand sheet positioned on top (**Figure 5** **, structures 12OP1-B to -E**). Single sheets, with 11 or 12 strands also appear with a frequency of 23.3% below melting (**Figure 5** **, structure 12OP1-A**). Surprisingly, strand orientation probabilities vary significantly going from the 3-peptide to the 12-peptide system. As for the 3-peptide system, the anti-parallel orientation is favored below melting for the 12-peptide system especially at the lowest two temperatures where the probability of forming anti-parallel is between 60% and 45% compared to 30% for the parallel. Then, as the temperature is increased, the amount of parallel and anti-parallel orientation becomes almost the same, suggesting that while anti-parallel orientation is energetically favored, it is rapidly overcome by the entropic gain of mixing orientations. The alignment of the β-strands is a mix of perfectly aligned strands and strands misaligned by one residue at all temperatures below the melting point. Because sheets are longer than for the trimer, the 12-mer comprises mostly β-sheets with strands in mixed orientations at low temperatures below T_m_ with a low probability of forming fully parallel or fully antiparallel sheets (**Table 2**). Interestingly in the few and much smaller sheets observed just above T_m_, fully parallel and antiparallel β sheets form with almost identical probability (data not shown), suggesting that with slower growth, structures visited below T_m_ could be more ordered.

#### All-atom MD simulations

The 5 most representative structures obtained from OPEP REMD (labeled 12OP1-A to 12OP1-E) were further studied by all-atom MD. Representative structures obtained from the latter simulations are shown in Figure 5 right panel. The 12OP1-A OPEP structure is characterized by the presence of a flat arrangement of β-sheets. It undergoes significant rearrangements during the all-atom evolution in explicit solvent (12GR1-A), as shown by the time evolution of the radius of gyration (**Figure S1**), with the planar β-sheet breaking into four fragments of two to four stranded β-sheets that assemble on top of each other, with two central parallel β-sheets covered on both sides by a perpendicular β-sheet. The overall amount of β-sheet structure is conserved during the all-atom simulation (**Table 3**).

**Table 3:** Comparison of the structural properties between OPEP and GROMACS structures for selected stable structures.

			β-Sheet Contenent (based on the dssp program)(b)				% Parallel - Antiparallel(c)				% Res Align (% 100 Align - % Shift by 1 Res - % Shift by 2 Res…)(d)			
			GROMACS - GR		OPEP - OP		GROMACS - GR		OPEP - OP		GROMACS - GR		OPEP - OP	
	Configuration Type(a)	Structure	First Cluster	Final Structure	CG	Min	First Cluster	Final Structure	CG	Min	First Cluster	Final Structure	CG	Min
Trimers	3	03_1 - A	38%	19%	71% (100%)	67%	50 - 50	50 - 0	50 - 50	50 - 50	50 - 50	50 - 50	50 - 50	50 - 50
	3	03_1 - B	38%	24%	19% (27%)	67%	0 - 50	50 - 50	50 - 50	50 - 50	50 - 50	50 - 0 - 50	50 - 50	50 - 50
	3	03_1 - C	57%	62%	62% (87%)	67%	50 - 50	50 - 50	50 - 50	50 - 50	50 - 50	50 - 50	50 - 50	50 - 50
	3	03_1 - D	48%	57%	52% (73%)	67%	0 - 50	0 - 50	0 - 50	0 - 100	50 - 50	50 - 50	50 - 50	50 - 50
	3	03_1 - E	48%	52%	57% (80%)	53%	50 - 50	0 - 50	0 - 50	0 - 100	50 - 0 - 50	50 - 0 - 50	0 - 50 - 50	0 - 50 - 50
12-mers	12	12_1 - A	49%	35%	64% (90%)	60%	60 - 20	40 - 10	36 - 64	36 - 64	80 - 20	50 - 50	64 - 18 - 18	64 - 18 - 18
	6 4 1	12_1 - B	14%	14%	45% (63%)	44%	13 - 25	20 - 40	22 - 22	33 - 33	35 - 25 - 37	0 - 40 - 60	33 - 56 - 11	33 - 56 - 11
	8 4	12_1 - C	35%	29%	49% (68%)	42%	10 - 20	40 - 20	60 - 40	40 - 30	40 - 40 - 20	50 - 20 - 30	60 - 20 - 20	60 - 20 - 20
	7 5	12_1 - D	33%	24%	54% (75%)	55%	11 - 22	0 - 30	40 - 60	10 - 60	45 - 44 - 11	50 - 50	50 - 50	50 - 50
	7 5	12_1 - E	56%	51%	46%	51%	60 - 40	50 - 20	60 - 40	60 - 40	60 - 30 - 10	50 - 50	60 - 30 - 10	50 - 40 -10
20-mers	11 7 1 1	20_p - A1	43%	38%	38% (53%)	37%	44 - 6	56 - 13	76 - 24	44 - 25	56 - 31 - 13	63 - 19 - 18	34 - 33 - 33	44 - 37 - 19
		20_p - A2	31%	34%	38% (53%)	37%	33 - 0	50 - 0	76 - 24	44 - 25	40 - 40 - 20	63 - 31 - 6	34 - 33 - 33	44 - 37 - 19
	11 7 1 1	20_p - B1	52%	49%	36% (50%)	66%	69 - 19	59 - 12	71 -24	75 - 25	31 - 44 - 25	35 - 47 -18	33 - 39 - 28	31 - 44 - 25
		20_p - B2	47%	38%	36% (50%)	66%	40 - 13	38 - 13	71 -24	75 - 25	34 - 53 - 13	31 - 56 - 13	33 - 39 - 28	31 - 44 - 25
	11 7 1 1	20_p - C1	39%	35%	44% (44%)	59%	44 - 19	47 - 20	72 - 22	69 - 25	31 - 50 - 19	54 - 33 -13	33 - 39 - 28	31 - 44 - 25
		20_p - C2	59%	36%	44% (44%)	59%	69 - 25	69 - 8	72 - 22	69 - 25	27 - 47 - 26	46 - 54	33 - 39 - 28	31 - 44 - 25
	12 7 1	20_p - D1	44%	46%	44% (62%)	64%	24 - 35	24 - 35	35 - 47	41 -47	59 - 35 - 6	65 - 33 -6	59 - 35 - 6	59 - 35 - 6
		20_p - D2	27%	27%	44% (62%)	64%	29 - 24	22 - 22	35 - 47	41 - 47	63 - 31 - 6	61 - 33 -6	59 - 35 - 6	59 - 35 - 6
	9 6 5	20_2 - A	44%	44%	34% (47%)	28%	69 - 13	61 - 11	72 - 22	73 - 13	31 - 44 -25	39 - 39 - 22	56 - 39 - 5	20 - 53 - 27
	8 7 5	20_2 - B	45%	39%	39% (55%)	71%	41 - 41	17 - 22	71 - 18	41 - 59	41 - 18 - 41	56 - 28 - 16	35 - 53 - 12	41 - 24 - 35
	7 7 6	20_2 - C	36%	37%	52% (73%)	65%	44 - 17	41 - 35	76 - 24	59 - 41	50 - 39 - 11	35 - 47 - 18	30 - 41 - 29	41 - 47 - 12
	8 8 4	20_2 - E	32%	33%	51% (72%)	63%	28 - 11	42 - 16	59 - 41	50 - 39	44 - 39 - 17	42 - 37 - 21	54 - 23 -23	39 - 33 - 28
	10 8 2	20_2 - N	41%	39%	42% (59%)	57%	39 - 22	41 - 24	31 - 56	31 - 56	44 - 28 - 28	29 - 47 - 24	44 - 25 -31	44 - 25 - 31

“First Cluster” means the most representative structure of the GROMACS simulations. “Final structure” is the final conformation obtained at the end of the GROMACS simulations. “CG” is the structure extracted at the end of the OPEP simulations before the reconstruction of the side chains. “Min” indicates the structure resulting from the reconstruction of the side chains after a minimization step.

(a) The configuration type (as described in the OPEP Analysis and Structure Selection section).

(b) The average amount (percentage) of residues in a β conformation. For OPEP, the percentage in brackets has been calculated without taking the Glycines into account.

(c) The average amount (percentage) of parallel and anti-parallel strands in a structure. The sum of parallel and antiparallel strands in a structure does not always total 100% if the structure sees strands in an undefined orientation, i.e. attached by only one hydrogen bond.

(d) The average amount of strands in-register and out-of-register (by one residue).

Structure 12OP1-B is characterized by a mainly parallel twisted β-sheet, with four strands packed on top. This structure is not stable in the all-atom MD setting, simulation 12GR1-B, and evolves towards a compact globular structure as shown by the evolution of the radius of gyration in time (**Figure S1**). Interestingly, the external side of the final aggregate is lined with hydrophilic Asn and Gln side chains that provide favorable contacts with the solvent. No specific order is observed for contacts among these side chains, although some cases of interdigitation as seen in the final steric zipper are noticed. The interior of the final aggregate is lined with Tyr aromatic side chains.

Such a supramolecular organization of the peptides may be representative of one of the soluble intermediates on the pathway to fibril formation. Solubility is favored by the presence of hydrophilic side chains on the external surface of the aggregate. At the same time, the packing of the interior is not optimal, so that the resulting structure may not be in the most favorable arrangement to ensure lasting stability. Water can also access the interior of the globular aggregate, disrupting inter-strand hydrogen bonds, eventually favoring conformational changes.

Structures 12OP1-C and 12OP1-D are similar to 12OP1-B: the main difference is that four strand pack with their long axis almost perpendicular to the long axis of the extended β-sheet. The main difference between 12OP1-C and 12OP1-D is that the planes defined by the four strands have different inclinations with respect to the plane of the long extended β-sheet. In the all-atom MD setting — simulations 12GR1-C and 12GR1-D — these structures evolve to less globular, but more compact final arrangements than that observed above, with most of the Tyr side-chains in contact with the solvent (**Figure S1**). The exterior of the aggregates is lined with Asn, while the interior is more compact than for 12GR1-A and 12GR1-B and packed with the side-chains of Gln, that form a network of van der Waals and hydrogen bonding contacts.

Finally, structure 12OP1-E is characterized by two orthogonal twisted β-sheets. The OPEP structure is very stable: it does not undergo significant rearrangement during the all-atom MD, contrary to the previous cases, and the β-sheet content remains constant (**Table 3**). The oligomer is trapped in this conformation by the extensive contacts packing determined by the Tyr side chains in the two sheets. Moreover, the inter-sheet space is filled by Asn and Gln side chains. However no specific packing into the ordered steric zipper is evident.

**Table 3** recapitulates the conformational heterogeneity and plasticity of the 12-mer aggregates. As a general case, the presence of explicit solvent tend to condense OPEP-generated structures, at the expense of structured β-sheets and the associated parallel-antiparallel structure, strand alignment and register. It must be kept in mind, though, that MD simulations may be affected by sampling limitations associated with the short runs and the presence of solvent.

Overall, the combined results indicate that the configurational richness increases from the trimer to the 12-mer and that the critical nucleus has not yet been found. Though, the strands do not see much exchange between sheets as seen in **Figure 5**. While ordered 12-mers are energetically much more favorable than the trimers, entropic factors may be considered prevalent, favoring a wide variety of metastable structures. The presence of explicit solvent decreases significantly the stability of elongated β-sheets either by increasing the effective hydrophobic interactions or decreasing entropic gains, favoring rather more compact structures. Different molecular mechanisms may be responsible for the stabilization of different conformations, endowed with different solubility properties. Indeed, we have observed globular-like structures with an external region decorated with hydrophilic groups that may determine the oligomers to be soluble in aqueous solution. In contrast, more ordered structures with higher β-sheet content appear to expose more hydrophobic area to the contact with the solvent. In turn, the latter may recruit more monomers or preformed oligomers that can aggregate by the juxtaposition of hydrophobic surfaces. The observations on the 12-mer systems also underline the enormous structural diversity that characterizes the aggregation of amyloidogenic peptides, which is reflected at the macroscopic level in a high degree of polymorphism.

### Simulations of 20-mer Systems

Next, we turned to the study of 20-mers in order to assess the importance of the number of chains on the final supra-molecular organization and determine whether new structural motifs can emerge.

#### Coarse-grained simulations

**Figure 6:**
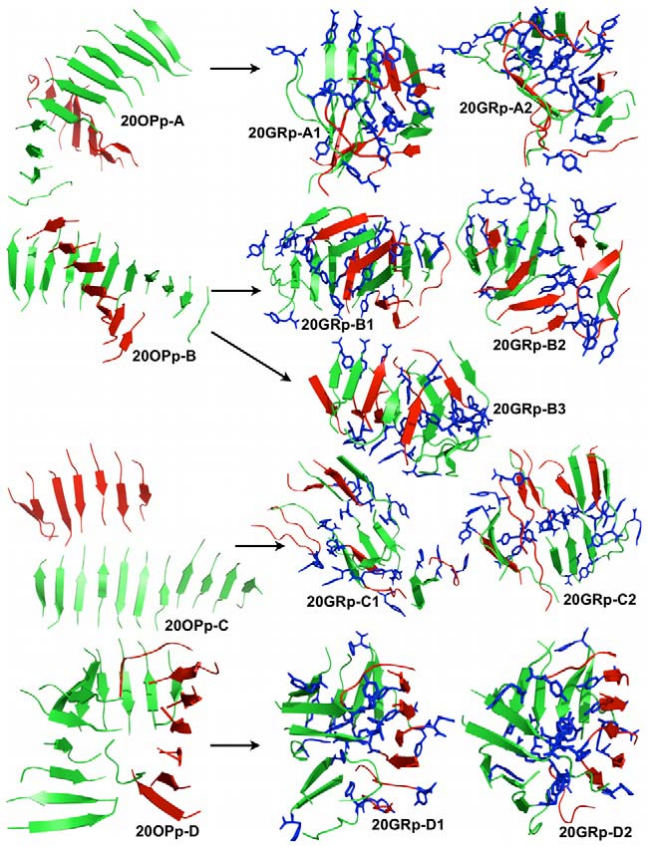
Structures obtained for the 20-mer preliminary simulations. The stable 20-mer structures obtained from OPEP's preliminary simulation 20OPp are shown on the left-hand side panel. The final primary clusters obtained from the OPEP structures with all-atom MD or all-atom REMD are displayed on the right-hand side panel. 20OPp-A,-B,-C and -D were extracted at 283.4 K. The color code is the same as in Figure 5. 20OPp-A is composed of 2 perpendicular β-sheets. 20OPp-B is a twisted β-sandwich fibril-like structure. 20OPp-C is made of 2 sheets on top of one another. 20OPp-D consists of a folded sheet (green) facing another shorter sheet (red). During the all-atom MD simulation the structures tend to be more globular with the strands seeing some exchange between the β-sheets, i.e. the red and green β-sheets from the OPEP structures dissociate and re-associate during the all-atom MD simulations except for structures 20GRp-D1 and -D2.

Three REMD simulations with OPEP were thus generated for the GNNQQNY 20-mer systems: 20OPp, 20OP1 and 20OP2. A preliminary run 20OPp was run to identify the four most common low-energy clusters, from which we extract the central structure for each: 20OPp-A, 20OPp-B, 20OPp-C and 20OPp-D (**Figure 6** **, left panel**). These were used as starting points for MD simulations with GROMACS. The first three are two-sheet structures while the fourth is a three-sheet configuration. What is particularly interesting here is that we obtain a protofibril-like structure (20OPp-B) among the most dominant clusters after only 200 ns starting from a random coil configuration. Interestingly, the protofibril-like structure is possible but not dominant in this preliminary simulation.

**Figure 7:**
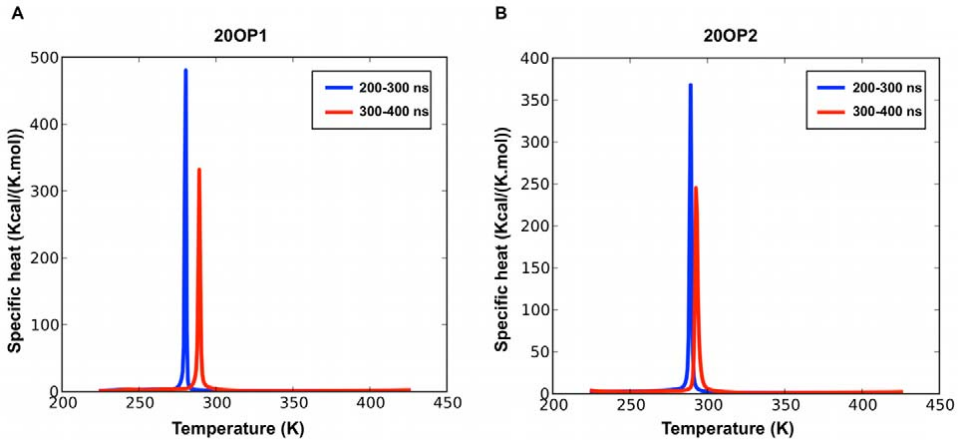
Specific heat as a function of temperature for the two 20-mer simulations sets. The specific heat is calculated over two time intervals for the systems 20OP1 (left panel) and 20OP2 (right panel) during the last 200 ns.

Following this preliminary run, we have performed two additional simulations 400 ns-long 20OP1 and 20OP2 (**Figure 7**) to attempt to better sample the phase space to determine the degree of preference and the importance of the protofibril-like structure among the morphologies accessible to that sequence for twenty peptides. Even after 400 ns, however, neither simulation is fully converged and the melting temperature is evaluated, from specific heat, to be at 280 K or higher, with ordered structures forming successfully below this temperature: the melting temperature is likely to continue to increase with the simulation length as the average nucleation time for the density used here appears to be around 1 µs based on the fact that slightly more than half the trajectories have not yet visited ordered structures during the 400 ns simulation. In spite of this limitation, we observe significant exchange among the trajectories below melting, suggesting that these achieve some degree of thermodynamic equilibrium.

As for the 12-mer, aggregation is extremely favorable energetically. The melting temperature for 20OP1 varies between 280.4 K and 289.2 K during the last 200 ns of simulation and the energy of ordered structures at the lowest temperature, 223.8 K, is on average −27.8 kcal/mol/monomer for 20OP1, as calculated from the PTWHAM analysis. For 20OP2, the transition is happening between 260.2 K and 290.5 K and the potential energy of aggregated structures at the lowest temperature, 223.8 K, is on average −28.1 kcal/mol, which is comparable to the energies of aggregated structures for 20OP1. Those energies are about 10 Kcal/mol/monomer above the dodecamer structures' energies at 222.5 K: clearly, the structures generated for the 20-mer are not as ordered as those found for the 12-mer due to the much longer time needed to sample these energetically-favorable conformations, but also because the entropic loss associated with full-ordering is larger for the 20-mer. For both the 20OP1 and the 20OP2 simulation sets, random coil structures dominate at simulations whose temperature is above 280 K.

**Figure 8:**
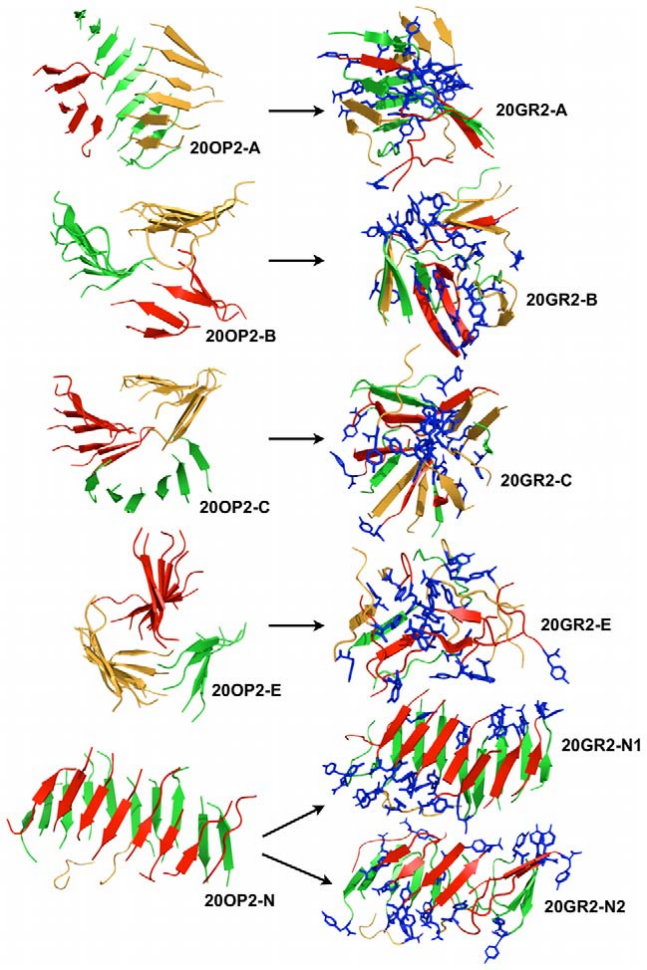
Structures obtained for the 20-mer 20OP2 and 20GR2 simulations. The final primary clusters obtained from the OPEP structures with all-atom MD or all-atom REMD are displayed on the right-hand side panel. 20OP2-A,-B,-C,-E and -N were extracted respectively at 260.1 K, 249.2 K, 254.7 K, 265.9 K and 292.2 K. The different sheets are distinguished by either a green, red or yellow color and the tyrosines are shown in blue sticks for the all-atom structures. Structures 20OP2-A,-B,-C and -E are composed of 3 sheets twisted around each other while structure 20OP2-N is a 2-sheet fibril-like conformation. During the all-atom MD simulation the structures tend to be more globular with the strands seeing some exchange between the β-sheets, i.e. the red, green and yellow β-sheets from the OPEP structures dissociate and re-associate during the all-atom MD simulations except for the fibril-like structures 20GR2-N1 and -N2.

Following specific trajectories, as they move through temperatures, it is possible to identify sequences of steps leading to low-energy ordered structures. In the more than 25 such events observed in 20OP1 and 20OP2, the aggregation process is systematically triggered by the formation of a few dimers, trimers and/or tetramers seeds. The conformations obtained from both 20OP1 and 20OP2 are structurally similar in the sense that they are almost always composed of three sheets composed of 5 to 9 strands each either facing each other in a triangle-like or organized in a propeller-like or β-sandwich conformation (**Figure 8**). Irrespective of the final shape, the system displays a strong tendency to form β-sheets. The five final ordered structures selected from 20OP2 and shown in **Figure 8** are representative of all three REMD simulation sets: below melting, the 20-chain system mostly forms three β-sheets, but can also form two-sheet structures. Looking at the statistics collected for 20OP1 and 20OP2 (**Table 4**), we observe that various three-β-sheet configurations with juxtaposed β-sheets containing 8-7-5 monomers or 9-6-5 monomers are frequent below T_m_. Two-β-sheet systems appear to be less frequent but are populated close to the melting temperature as well. Although, sheet-lengths differ slightly between simulations 20OP1 and 20OP2 for the dominant structures, the overall results are consistent. We also observe a high number of possible β-sandwich morphologies for the three-sheet configuration among the final structures obtained where some of them see their three sheets facing each other in a triangle or twisted-around-each-other arrangements (**Figure 8**, structures 20OP2-A, -B, -C and -E). These topologies run from a rather well defined 3-fold symmetry (20OP2-E) to more disordered conformations with little symmetry (20OP2-A, -B and –C). The minimal β-sheet unit contains four strands in 20OP2-E, five strands in 20OP2-A,-B and six strands in 20OP2-C. We note that the structure 20OP2-E is reminiscent of the recently proposed structure of Aβ1–40 fibrils with a three-fold symmetry [14]. Interestingly, ordered two-sheet conformations with one sheet slightly longer than the other, such as 11 9 and 12 8 (not shown, but close to 20OP2-N, a 8 10 2 configuration in Figure 8), represent a significant fraction of the accessible states either as a β-sandwich –with an occasional insertion of a short β-sheet – or as two perpendicular sheets.

**Table 4:** Structural characteristics for the 20-mer aggregates as a function of temperature below the melting point.

			Population							
			223.8 K	249.3 K	260.1 K	265.9 K	270.3 K	273.8 K	277.1 K	280.1 K
20OP1	Configuration types (%)^(a)^	8 7 5	93.2	12.4	7.5	7.1	9.8	6.8	7.1	9.4
		8 7 3 2	0.0	44.7	2.6	0.8	0.4	0.8	0.0	0.0
		7 7 6	2.6	12.4	13.9	9.8	12.8	10.5	12.4	7.9
		10 6 4	0.4	0.0	7.5	8.7	5.3	11.3	5.3	3.4
		11 5 4	0.0	0.8	7.5	6.4	6.0	3.4	1.5	0.0
		11 5 2 2	0.0	10.5	0.0	0.0	0.0	0.0	0.0	0.0
		14 6	0.0	0.8	5.3	6.8	7.1	7.5	3.4	4.9
		15 5	0.0	0.0	3.4	7.1	3.8	4.5	6.8	8.3
		10 10	0.0	0.4	4.5	8.7	6.0	5.3	7.9	2.3
		10 5 5	0.0	0.0	1.9	3.8	8.3	7.1	3.8	2.6
		11 9	0.0	0.0	3.8	9.0	11.7	6.8	7.1	4.5
		20	0.0	1.1	3.4	4.9	5.3	5.3	3.4	0.0
	% parallel^(b)^		73.9	48.8	53.4	55.1	56.2	56.8	58.4	46.8
	% antiparallel^(b)^		25.1	48.2	41.3	37.6	36.7	35.6	34.5	29.4
	% fully parallel sheets^(c)^		26.2	22.4	19.7	15.1	11.8	14.5	11.1	16.1
	% fully antiparallel sheets^(c)^		0.9	7.9	3.1	4.6	1.9	2.5	1.0	6.5
	% mixed sheets^(c)^		72.9	69.7	77.2	80.3	86.3	83	87.9	77.4
	β-sheet content (%)^(d)^		59.1	61.3	60.5	60.5	60.5	59.8	57.1	45.7
	% Strands in-register/out-of-register by 1 residue^(e)^		32.1/38.0	43.1/31.2	42.6/33.8	41.2/36.9	40.1/38.4	38.8/38.6	39.7/38.6	38.7/39.8
20OP2	Configuration types (%)^(a)^	9 6 5	29.3	29.3	24.4	24.1	18.4	15.4	8.7	8.3
		9 7 4	0.4	1.5	4.5	4.5	7.5	4.9	6.0	3.8
		8 8 4	1.1	0.0	1.9	1.1	3.8	6.0	4.5	4.1
		7 7 6	4.5	6.0	8.7	12.0	12.8	16.2	15.0	12.0
		15 5	12.4	9.4	7.5	2.3	6.8	3.4	1.9	1.5
		13 7	0.8	0.4	0.4	1.9	1.5	2.3	4.9	7.1
		14 6	3.4	3.8	3.0	4.9	10.9	6.4	8.7	6.4
		11 9	9.4	14.7	6.8	3.8	2.3	4.5	5.6	3.8
		11 5 4	16.5	12.4	3.8	0.0	0.4	1.5	0.0	0.0
		8 7 5	0.4	1.9	1.5	1.9	5.6	6.8	6.4	6.0
		10 10	0.0	0.0	0.0	0.0	0.0	0.0	0.0	0.0
		20	0.0	0.0	0.0	0.0	0.0	0.4	0.0	0.4
	% parallel^(b)^		53.1	56.6	57.1	56.8	56.7	55.7	56.7	55.1
	% antiparallel^(b)^		38.1	35.7	36.5	37.5	37.9	39.3	38.4	38.5
	% fully parallel sheets^(c)^		7.4	16.9	15.0	14.2	14.1	14.1	18.7	17.1
	% fully antiparallel sheets^(c)^		0.6	4.7	3.2	4.2	3.9	4.4	5.1	8.1
	% mixed sheets^(c)^		92.0	78.5	81.8	81.6	82.0	81.5	76.2	74.8
	β-sheet content (%)^(d)^		66.5	63.4	63.0	62.5	61.5	60.6	58.7	56.0
	% Strands in-register/out-of-register by 1 residue^(e)^		51.0/36.3	50.0/34.0	48.1/35.1	44.6/37.0	42.4/37.2	42.3/37.2	39.9/39.1	39.7/39.4

Temperatures above 280.1 K are not displayed here since they are populated essentially by conformations with random coil monomers with no secondary structure. The percentages are calculated over all the structures obtained in the last 200 ns of both OPEP REMD simulations. For details on ^(a)–(e)^, see Table 2.

In both REMDs, the β-sheets have a high probability of being in a mixed anti-parallel/parallel orientation state due to their length (over 70% below Tm) (**Table 4**). As for the dodecamer, as T rises, sheets become shorter and we notice a rise of fully parallel sheets at the five temperatures above T_m_ (data not shown). Contrary to the 3-mer and 12-mer where the β-strands prefer an anti-parallel orientation below 300 K, the parallel orientation of the strands is preferred in the 20-mer at all temperatures. The extended chains are also dominantly perfectly aligned at the lowest temperatures and the structures see a mix of perfectly aligned strands and strands misaligned by one residue close to the melting point. The dominance of perfectly aligned strands and parallel orientations of the chains is consistent with the experimental observations that GNNQQNY fibrils display parallel β-strands [9].

#### All-atom MD simulations of dominant clusters generated by OPEP-REMD 20OPp

Consistent with the protocols described above for the 3-mers and 12-mers, the dynamical properties of 9 selected oligomeric conformations generated by the OPEP simulations were refined by all-atom MD simulations in explicit solvent (see **Table 1**).

The first set of all-atom MD simulations was run on the structures selected from 20OPp calculations. OPEP runs identified two main types of 3D organization for the 20mer: extended β-sheet and globular-like structures. The former are characterized by the presence of two parallel sheets, while the latter are characterized by a circular organization of the strands, in a mostly parallel arrangement. The major representatives of the extended β-sheet like structures obtained from OPEP are labeled 20OPp-B and 20OPp-C; the globular structures are recapitulated by 20OPp-A (which shows a compact part packed by a more extended sheet) and 20OPp-D, see **Figure 6**. Two sets of 100 ns MD simulations, starting with different initial velocities, were run for each structure.

Aggregates with facing B-sheets. The structure of 20OPp-B is characterized by the presence of two perpendicular β-sheets. Each β-sheet consists mainly of twisted parallel strands.

After the first MD run (20GRp-B1) the two sheets are oriented anti-parallel to each other, forming a tight and elongated structure (**Figure 6**). The Asn side chains from opposite sheets occupy the inter-sheet space with some intertwining of the amide side chains. Significant packing is also provided by the aromatic rings of Tyrosine belonging to adjacent strands in the same sheet. The 100-ns generated aggregate also displays a significant degree of twisting in the strands that make up the two facing antiparallel β-sheets. This final structure is similar to that observed by others [50]. It is important to underline that this twisted configuration forms spontaneously during the MD simulation time starting from a less compact structure.

**Figure 9:**
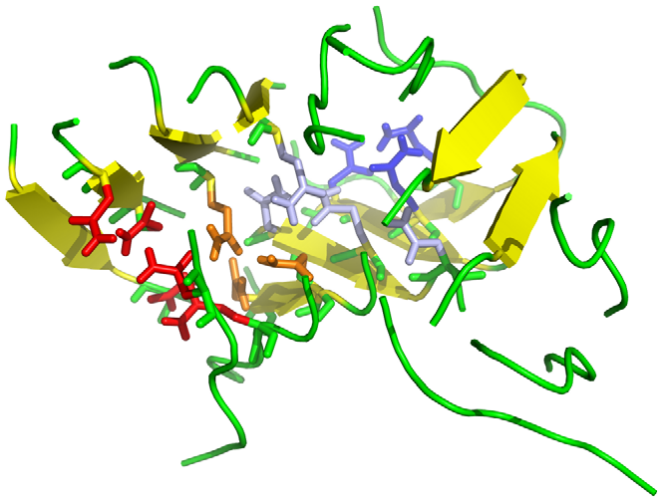
Structure 20-mer showing interdigitation of the side-chains. This structure is representative of the 20GRp-B2 simulation. It shows the inter-sheet space filled by Asn and Gln side chains, which in some case interdigitate.

In the second MD simulation (20GRp-B2), starting from the same initial structure with a different set of velocities, the β-sheet content decreases due a lower degree of packing of the Tyr side chains and interdigitation of the Gln and Asn side-chains (**Figure 9**). Packing interactions, however, still appear to be important in stabilizing the compact structure.

During both 20GRp-B1 and 20GRp-B2 MD simulations, the strands are dynamically interchanged between the two β-sheets.

Two elongated sheets may, however, evolve towards very different supramolecular organizations. In 20OPp-C two elongated β-sheets, with mainly parallel β-strands, are in contact through the terminal Tyr aromatic chains in an extended and non-compact structure (**Figure 6**). In both all-atom MD simulations, 20GRp-C1 and 20GRp-C2, the two β-sheets break up and reorganize in more compact conformations. Though, for 20GRp-C2, the strands do not interchange between sheets while 20GRp-C1 sees some mixing between the strands of the 2 β-sheets. The compact final structures still show some of the features we have described in the previous case: anti-parallel orientations of facing sheets, compaction of the side chains. One part of the long parallel β-sheet, represented by a four-stranded unit, detaches from the initial complex and packs onto the compact structure described above, minimizing the exposed surface area. The resulting structures correspond to a β-sandwich composed of three sheets. The time evolution of the radius of gyration is reported in (**Figures S2, S3**).

Evolution of globular structures. All-atom MD simulations were also started from more densely packed structures obtained from OPEP runs, represented by 20OPp-D and 20OPp-A. Each starting structure was subjected to two 100 ns MD simulations, resulting in simulations 20GRp-A1, 20GRp-A2, 20GRp-D1 and 20GRp-D2 (**Figure 6**). In the case of 20OPp-D both all-atom MD runs show that the starting conformation retains its overall structure with mostly parallel β-strands. Most of the Tyr side chains form a compact hydrophobic core at the interior of the structure. During all-atom MD evolution they reorganize and form small compact clusters that are optimally packed through pi-stacking face-to-face interactions, minimizing unfavorable contacts with the solvent. In general, the interior of the aggregate is compact and hydrophobic while the external surface is decorated with the more hydrophilic amide side chains. From the biochemical point of view, this structure could be described as a “soluble” oligomeric state reminiscent of the ones observed for the 12-mer run.

Starting from 20OPp-A, simulation 20GRp-A1 evolves towards a compact globular structure, in which parts of the ordered β-sheets are lost and strands are dynamically interchanged between sheets. The aromatic Tyr side chains cluster in the hydrophobic core of the structure and Asn side chains align on the surface. Most of the conserved sheets are still in parallel orientation.

Strikingly, in simulation 2 (20GRp-A2) the initial structure evolves to form two twisted antiparallel sheets in which the constitutive strands are parallel to each other. This structure resembles the twisted conformation observed for 20OPp-B and was observed in previous simulations [50]. The inter-sheet space is filled by Asn and Gln side chains, which in some case interdigitate (**Figure 9**). The conformation of this intermediate still shows some Tyr aromatic side chains in the interior, disrupting the optimal interdigitation of amidic side chains and causing the structure to be non-perfectly ordered (**Figure 6**).

These results suggest that the sheet organization in the twisted antiparallel conformation(s) may be accessible on the aggregation pathway, once two sheets are formed and docked upon each other. Interestingly, we have observed the formation of elongated, twisted antiparallel structures in MD only in the 20-mer system. The latter appear to evolve preferentially towards globular structures, suggesting that elongated, fibril-like conformations of the oligomers may be accessible only in the presence of a higher number of monomers. At the atomic level, sheet-locking is favored by the packing of Asn and Tyr side-chains. The Tyr aromatic packing and the initial formation of steric-zipper-like structures also provide important contributions in determining the ordering and stabilization of the growing aggregate and, possibly, its evolution to a stable fibril.

#### All-atom MD simulations of the dominant clusters generated by OPEP-REMD 20OP2

The detailed role of side-chains in determining the conformational characteristics of compact aggregate structures was further evaluated by analyzing at atomic resolution a set of diverse OPEP structures: 20OP2-A, 20OP2-B, 20OP2-C, 20OP2-E and 20OP2-N (**Figure 8**).

The starting structures of the MD simulations from 20OP2-A, 20OP2-C and 20OP2-E of the aggregates all consist of three extended β-sheets, organized in different tertiary arrangements (see **Figure 8**). The all-atom evolution of structure 20OP2-A leads to a more compact and globular-like conformation with a mixing of the β-strands and a partial loss of ordered β-structure involving external strands (**Figure 8** **, structure 20GR2-A**). Tyr side-chains mainly pack in the interior of the globule in the representative structures of MD simulations while most of the hydrophilic side chains (Asn and Gln) are located on the exterior of the oligomer pointing towards the solvent. This arrangement would confer water solubility to the oligomer by sequestering hydrophobic-aromatic side-chains to the interior of the aggregate and decorating the external surface with hydrophilic groups.

In the case of 20OP2-C, the starting structure constituted by three β-sheets, which are lined and twisted along a common axis, is not stable in the all-atom MD setting, and immediately evolves to a more compact globular structure that however does not display specific supramolecular properties or preferential orientations of the strands within the aggregate (**Figure 8** **, structure 20GR2-C**).

In the case of 20OP2-E, the evolution of all-atom MD simulations at 300 K determines a large decrease in the degree of ordered β-structure leading to the formation of disordered, amorphous conformations (**Figure 8** **, structure 20GR2-E**).

The remaining two representative clusters obtained from OPEP simulations display different three-dimensional arrangements. In the case of 20OP2-B, the structure is characterized by parallel β-sheet motifs that form a less compact conformation than the one observed above. All-atom MD evolution leads to a globular structure with a global reorganization of the β-strands (**Figure 8** **, structure 20GR2-B**). A large number of Asn and Gln amidic side-chains point towards the interior of the globular structure, and a number of hydrophobic Tyr side-chains are aligned in contact with the solvent. The reorganization of the starting structure to this globular intermediate determines a partial loss of ordered secondary structure in some of the constituent peptides. This structure retains a large hydrophobic surface in contact with the solvent. The large hydrophobic area exposed to water may be one of the causes of the insoluble character of these intermediates.

Finally, we simulated the structure of cluster 20OP2-N at all-atom resolution as this aggregate forms an elongated structure with two facing β-sheets. MD evolution at 300 K for this system shows no reorganization of the β-strands (**Figure 8**, structure 20GR2-N1). In contrast to what we observed for 20OP2-B, the Asn and Gln amidic side-chains fill the space between the two sheets, establishing H-bonding interactions, and showing interdigitation of side-chains reminiscent of the experimentally observed dry steric zipper. The sheets are not all perfectly parallel, and this might oppose the formation of a perfectly packed steric zipper.

Summarizing, as shown in **Table 3**, the structural properties of the aggregates cannot be easily rationalized into specific classes: great variability is observed in terms of β-sheet content, percentage of parallel vs. antiparallel arrangements, register and relative orientations of the strands. Interestingly, it appears that the fibril-like structure 20GR2-N1 is stable during the 100-ns all-atom MD simulation, while the other arrangements undergo large reorganization. In general, the consistency of the results in terms of conformational plasticity for all constructs clearly indicates that a wide range of different structures is accessible to relatively large sized oligomers.

#### All-atom REMD simulations of two elongated 20mer structures generated by OPEP-REMD

The all-atom simulations of 100 ns starting from 20OPp-B and 20OP2-N showed the possibility for the aggregates to remain elongated partially ordered oligomers whose structures are reminiscent of the arrangements observed by X-rays of micro-crystals. In order to gain more insights into the stability and conformational evolution properties of these structures, we set out to run all-atom REMD simulations starting from OPEP structures 20OPp-B (**Figure 6**) and 20OP2-N (**Figure 8**). The resulting all-atom REMD simulations are labeled 20GRp-B3 and 20GR2-N2, respectively.

**Figure 10:**
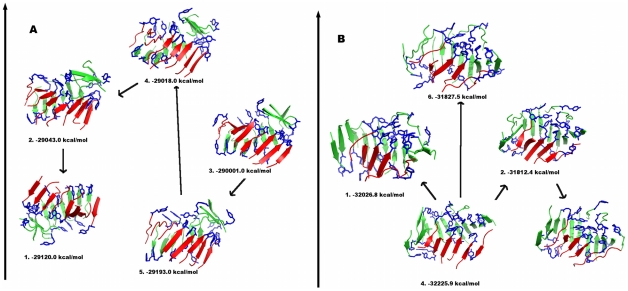
Representative structures of the most populated clusters from all-atom REMD simulations of the 20mer: (a) structure 20GRp-B3 and (b) structure 20OP2-N. The number identifying each structure represents the cluster rank (1. being the most populated cluster). The value of the GB/SA energy in water of the complex is reported. Arrows represent transitions between clusters, indicating possible paths between cluster structures.

In the REMD simulation labeled 20GRp-B3, and similarly to what is seen in OPEP REMDs, we observe that structures interconvert between compact and elongated conformations with a pair of sheets facing each other. The main representative structures for simulation 20GRp-B3, and their relative stabilities, are reported in **Figure 10a**. In the elongated conformation, the relative orientation of the strands within the sheets tends to be parallel. The two facing strands are oriented antiparallel to each other. The Tyr aromatic side chains form clusters of packed rings that are reminiscent of the arrangements observed in the crystals from the Eisenberg group (**Figure 10a**). Partial ordering of the Gln and Asn side chains into the zipper spine arrangement is also observed. This conformation, labeled as conformation 3 in **Figure 10a**, is however not stable enough to be the most populated structure at the lowest temperatures. Representative structures of the most representative clusters are extracted from the trajectories and their relative free energies evaluated with the GB/SA approach implemented in the program MacroModel, and according to what was already reported [44]. These calculations provide an approximated energetic value for the stability of the aggregates in solution, and show that the more elongated structures tend to undergo transitions to more globular like conformations.

Several conformational transitions among different structural clusters are observed, and highlighted by direction arrows in **Figure 10a**. Summarizing, the starting conformation for 20GRp-B3 with two facing sheets antiparallel to each other shows a high tendency to interconvert rapidly into globular, compact structures. The high conformational plasticity and limited stability of elongated conformations had already been noticed in the present OPEP-based simulations. Analysis of the time evolution of REMD trajectories shows that globular conformations form by detachment and re-docking of β-sheet motifs. During the formation of the more globular structures a slight decrease of the amount of ordered β-sheet could also be noticed.

All-atom REMD simulations were also used to analyze the structural evolution of cluster 20OP2-N. The representative structures obtained from the all-atom simulation 20GR2-N2 are reported in **Figure 10b**, with their relative stabilities. The packing of the interior of the initial aggregate is not optimal. Consistently with the previous case, the overall aggregate still shows a high degree of structural plasticity undergoing transitions to conformations with a lower degree of ordered β sheet and more globular-like shapes. Elongated structures featuring two facing β-strands still appear and show partial interdigitation of Asn and Gln side chains in the inter-strand space, with formation of a steric zipper motif. This does not seem sufficient, however, to provide the necessary stabilization for the aggregate to evolve to a protofibril structure. Once more, a number of transitions among different structural families (**Figure 10b**) are observed.

Overall, we observe that the elongated structures reminiscent of the one observed by the Eisenberg group in the fibril microcrystals is accessible at room temperature but is not stable and can easily interconvert into globular or more disordered conformations, even in the presence of 20 monomers (see **Table 3**). According to our observations, this happens both by detachment-reattachment of strands or small oligomers from existing structures, or through sliding/reptation moves without detaching from the aggregates [81], [82]. This behavior is widely observed across our 20-mer simulations while the 12-mer system does not undergo much reorganization during all-atom MD simulations. Interestingly, when ordered supramolecular structure forms, with either an elongated or a compact globular shape, there is no space for water in the interior of the aggregate. Water exclusion from the core of the aggregate may represent the first step leading to the formation of a dry interface.

### Conclusions

The self-organization process of peptides and proteins into oligomeric soluble and insoluble aggregates that eventually evolve to fibrils is still difficult, if not impossible, to study at atomic resolution using experimental approaches. In this paper, we have carried out an extensive and comprehensive study of the formation of oligomers of the model peptide GNNQQNY in different conditions combining coarse-grained and all-atom simulation approaches. Different numbers of peptides were used in several simulations. In the smallest systems, composed of three strands, a diverse set of structural motifs is accessible at room temperatures. When bigger systems consisting of 12 chains are analyzed, compact and globular structures begin to appear. Interestingly, in some cases, globular oligomers expose hydrophilic side chains to the contact with the water solvent, providing a viable model for soluble intermediates that have been observed on pathway to the formation of the final fibril. In parallel, at 300 K, globular structures exposing a large amount of hydrophobic surface also appear. These may represent possible nuclei for the growth of bigger supramolecular structures.

In simulations conducted using 20 monomers, we have noticed the appearance of elongated structures characterized by the juxtaposition of two mainly parallel β-sheets with partial interdigitation of amidic side chains reminiscent of the zipper-spine observed in fibril microcrystals. It is important to notice, however, that these structures are not stable in water solvent and evolve towards more globular conformations. This observation suggests that while ordered fibril-like structures are accessible on the energy landscape, they need further stabilization by establishing contacts with multiple copies of similar structures in order to evolve to a fully fibrillar geometry. In this context, the formation of this geometry would require the constructive interplay of many factors and the entropic expense of such process would be clearly very high, explaining the long lag phase times and very slow kinetics of amyloid fibril formation. Moreover, the rich variety of structures and conformational changes observed for the aggregates may also reverberate into the fibril polymorphism observed at the experimental scale.

In summary, our data and structural models represent valid complements to experimental approaches in the attempt to shed light on the supramolecular arrangements of amyloidogenic oligomers, and lead the following conclusions.

First, the 20-mers of GNNQQNY are polymorphic and endowed with a high degree of structural plasticity. Polymorphism of the fibrillar products of amyloid aggregation has been observed for many sequences by X-ray diffraction and solid-state NMR experiments [6], [14], [21] and a recent computational study using a simplified description, where the peptide has a single degree of freedom with two minima [83]. Polymorphism of the low-n oligomers with different registers of inter-peptide H-bonds and orientations of the chains has also been reported using various force fields and computational procedures [22], [41], [44], [46], [84].

Second, the 20-mers of GNNQQNY in explicit water are in dynamic equilibrium, within at least 100 ns, between amorphous structures (high probability) and configurations with three β-sheets in various orientations (medium probability) and two β-sheets (low probability). These two-β sheets, reminiscent of the cross-β structures and the dry steric zipper observed experimentally for mature fibrils, are not parallel, however, suggesting the existence of a free energy barrier preventing the formation of a perfectly packed steric zipper.

Third, there is a reorientation of the β-strands between the GNNQQNY oligomers and fibrils. We find that an anti-parallel β-strand alignment dominates over the parallel one in the 3 and 12 peptide systems. This contradiction with the fibrillar parallel β-strand orientation [7] is however reconciled by the 20-peptide systems, where a significant increase in the amount of parallel strands and fraction of fully parallel β-sheets is observed.

Fourth, a common observation is that short amyloid peptide fragments assume antiparallel β-strand geometries whereas longer peptides, and proteins, often assume parallel geometries. Our simulations along with other recent studies show this geometrical property is more complex and depends strongly on the amino acid composition. The dependence of β-strand orientation with oligomer size occurs in the GNNQQNY (Sup35) peptide and the VQIVYK (PH6) peptide, as reported by another computational study [85], because both short peptide fragments do not contain any opposite charged amino acids at the extremities as opposed to numerous fragments, such as the KLVFFAE (Aβ(16–22)) and the KFFE peptides. Table 2, for example, shows that the free energy difference in favor of the antiparallel sheet decreases from 0.70 kcal/mol for the trimer to only 0.05 kcal/mol for the dodecamer at 300 K. This competition between the two β-strand orientations during polymerization is also supported experimentally by the co-existence of either antiparallel or parallel strands in seeded hIAPP20–29 (SNNFGAILSS) fibrils [15], and the fact that D23N-Aβ40 forms fibrils with a majority having antiparallel β-sheet structures and a minority having parallel β-sheet structures [86]. We hypothesize that both short polar peptides and longer peptides could display this β-strand orientation transition during aggregation.

In addition, antiparallel β-sheets allow a higher potential variability of the inter-chain H-bond geometry [87], [88] accommodating a higher number of possible strand conformations, which is the favored situation in smaller aggregates. A parallel organization could be more favorable for larger aggregates, which can for instance create double-layered sheets that can pack with a parallel arrangement of the strands as indicated by SS-NMR [89]. Moreover, in larger aggregates, the parallel arrangement might be stabilized by the definition and stabilization of large numbers of side-chain side-chain contacts. Therefore, our results are in agreement with experiments but also suggest a more complex relationship between the monomers during the assembly process, something that cannot be measured experimentally.

Fifth, in terms of experimental relevance, it is important to note that evidence exists showing that aggregation pathways can be manipulated by the use of molecular chaperones. In the case of the Sup35 prion protein, the chaperone Hsp104 catalyzes the polymerization of seeds that are crucial for efficient amyloid formation [90]. Different chaperones such as Hsp70 and Hsp40, on the contrary, prevent the assembly of aggregating species when added during the polymerization reactions. In these cases, soluble aggregates are formed, showing that chaperones can redirect amyloidogenic polypeptides into non-amyloidogenic species. Finally, the chaperonin TRiC has also been shown to be a modulator of amyloid formation [91]. TRiC, in combination with Hsp70 and 40, stimulates the reassembly of huntingtin oligomers into soluble species, which are non-toxic. Clearly, the type of binding and structural remodeling determined by different chaperones is dictated by the details of the molecular recognition between the oligomers and the interaction surfaces of the chaperones. In this context, it is worth noting that generating plausible models of possible oligomeric substrates for the chaperones may be of great help in the design of optimized systems aimed at modulating aggregate properties. Based on the structural and surface properties of the oligomers, models of their complexes with chaperones may be generated.

On this basis, the hydrophobic-hydrophilic profile of the chaperone interaction surfaces could, for instance, be changed by means of site-directed mutagenesis, affecting their activity and ultimately the properties of the remodeled oligomers. This would allow a rational manipulation of the amyloidogenic pathways, helping to shed light on a very complex biological phenomenon.

A final consideration helpful to put our results in a biological perspective is related to the importance of the knowledge of oligomeric structures in the design of amyloidogenic inhibitors. In this context, we are currently exploring the characterization of the solvent accessible hydrophobic surface area of the 20-mers to guide docking-experiments of small-molecule compounds (Congo Red and EGCG in particular), in order to derive possible rules for the rational selection of aggregation inhibitors. Preliminary data and results show that this could be helpful in alleviating the difficulties associated to drug-design when dealing with amyloid-targets. Indeed, compared to classical drug-design efforts where the target is an active site, with well-defined structure and cavities, the variety of structures, mechanisms and conformational plasticity of oligomers shown here confirm that rational design of aggregation inhibitors is a daunting challenge. However, careful characterization of oligomeric structures provides useful suggestions for the design of possible inhibitors. Selective compounds or peptidomimetics could be designed/selected to target the oligomer conformations characterized by the presence of aromatic groups on their external surface. These compounds would actually target intermediates that are more prone to be insoluble or to favor the addition of monomers through hydrophobic interactions. Interestingly, most of the existing inhibitors of amyloidogenic pathways are small molecules rich in aromatic functionalities, which can target more than one single aggregating species, showing a general mechanism of action [92].

Alternatively, one could design peptidomimetic-based or small molecule chaperones that can stabilize soluble species, subtracting them from the amyloidogenic pathway. This would lead to a redirection of otherwise amyloidogenic peptides into non-amyloidogenic species.

## Supporting Information

Figure S1 Time evolution of the radius of gyration of the 12-mer oligomers. From top to bottom: structures 12GR1-A, 12GR1-B, 12GR1-C, 12GR1-D and 12GR1-E. The structures shown are the final structures of the all-atom MD simulations with GROMACS.
(TIF)

Figure S2 Time evolution of the radius of gyration of the 20-mer oligomers for the preliminary simulation. Structures 20GRp-B1, 20GRp-B2, 20GRp-C1 and 20GRp-C2. The structures shown are the final structures of the all-atom MD simulations with GROMACS.
(TIF)

Figure S3 Time evolution of the radius of gyration of the 20-mer oligomers for the preliminary simulation. Structures 20GRp-A1, 20GRp-A2, 20GRp-D1 and 20GRp-D2. The structures shown are the final structures of the all-atom MD simulations with GROMACS.
(TIF)
