# Supplementary figures and images for: A Multiscale Approach to Characterize the Early Aggregation Steps of the Amyloid-Forming Peptide GNNQQNY from the Yeast Prion Sup-35
Source: PLoS Comput Biol. 2011 May 19;7(5):e1002051. doi: 10.1371/journal.pcbi.1002051 (PMC3098217; doi:10.1371/journal.pcbi.1002051)

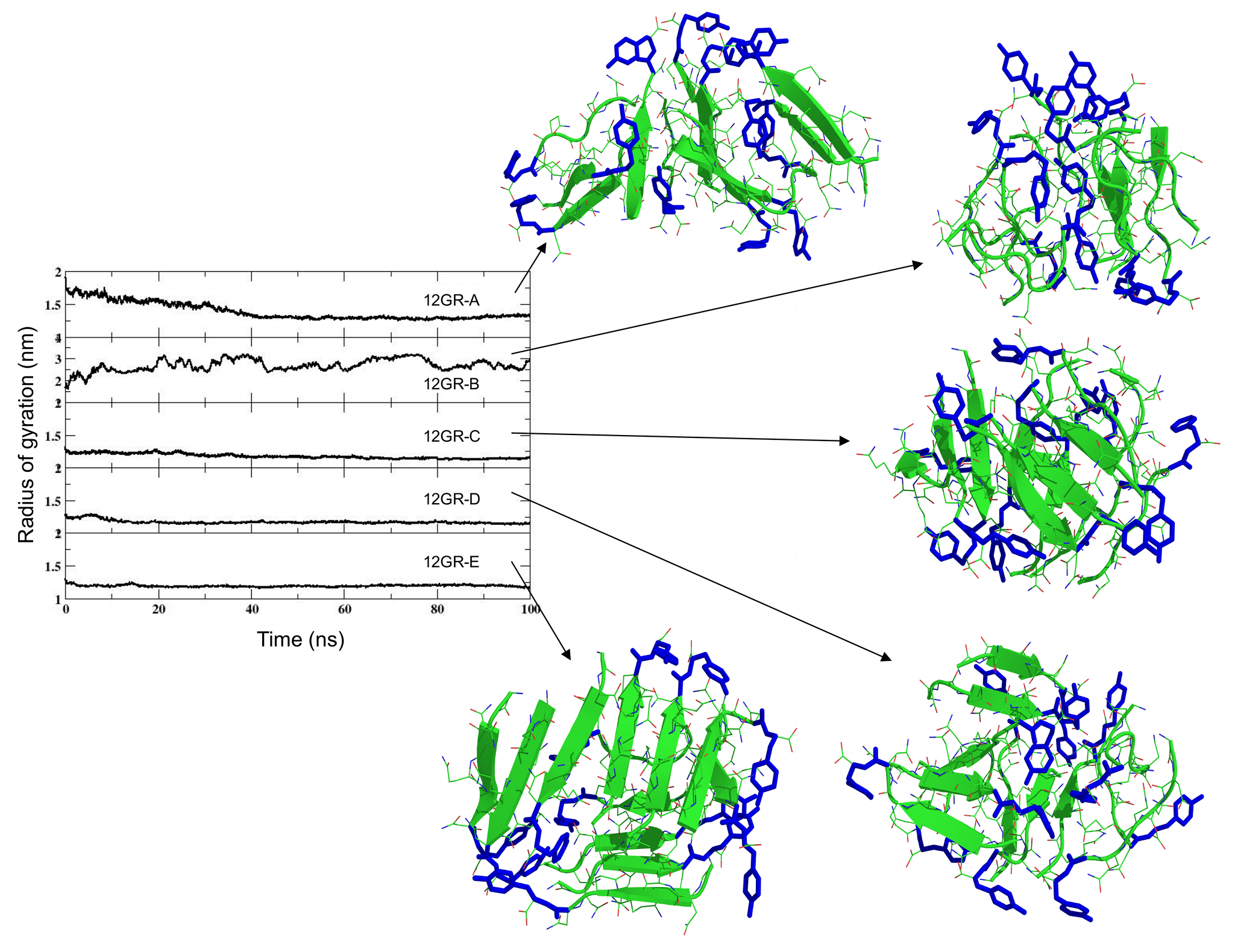

Supplement: Figure S1 — Time evolution of the radius of gyration of the 12-mer oligomers. From top to bottom: structures 12GR1-A, 12GR1-B, 12GR1-C, 12GR1-D and 12GR1-E. The structures shown are the final structures of the all-atom MD simulations with GROMACS. (TIF) [file pcbi.1002051.s001.tif]

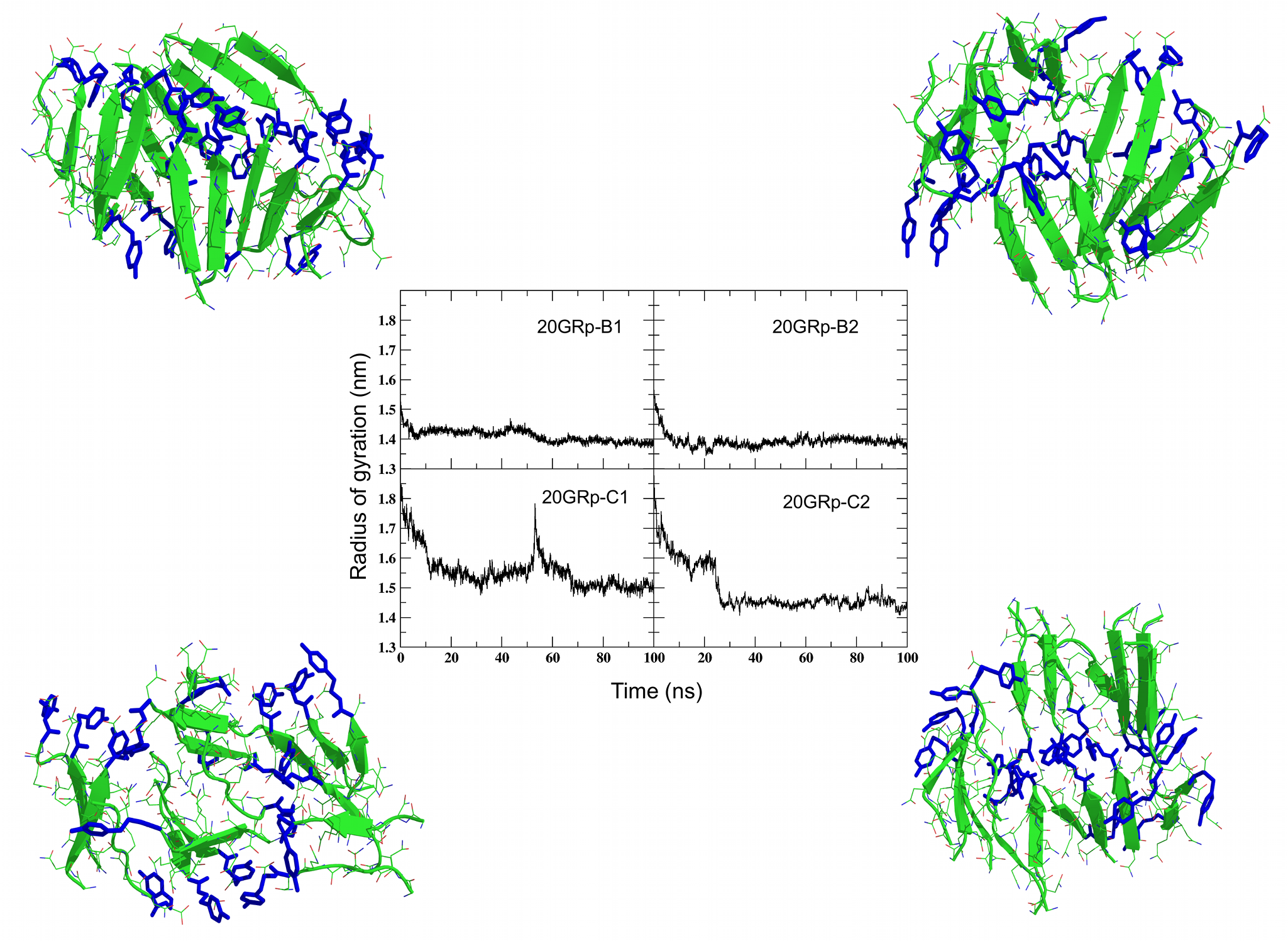

Supplement: Figure S2 — Time evolution of the radius of gyration of the 20-mer oligomers for the preliminary simulation. Structures 20GRp-B1, 20GRp-B2, 20GRp-C1 and 20GRp-C2. The structures shown are the final structures of the all-atom MD simulations with GROMACS. (TIF) [file pcbi.1002051.s002.tif]

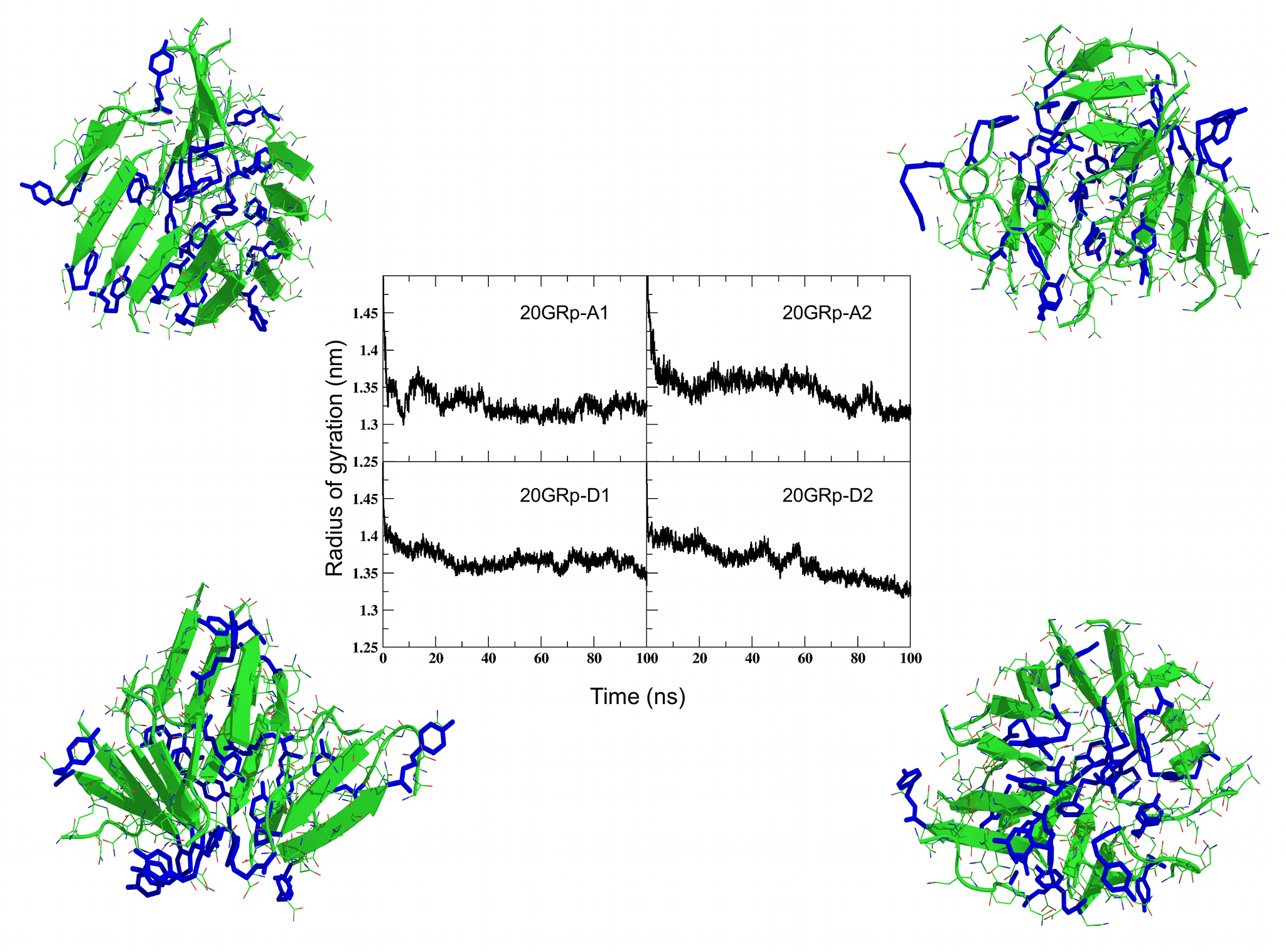

Supplement: Figure S3 — Time evolution of the radius of gyration of the 20-mer oligomers for the preliminary simulation. Structures 20GRp-A1, 20GRp-A2, 20GRp-D1 and 20GRp-D2. The structures shown are the final structures of the all-atom MD simulations with GROMACS. (TIF) [file pcbi.1002051.s003.tif]
